# Impact of Sample Storage Conditions on Phenolic Profiles of Commercial Wines Assessed by UHPLC-HRMS: Implications for Analytical Reproducibility

**DOI:** 10.3390/molecules31213867

**Published:** 2026-10-27

**Authors:** Nahikari Prevost, Alan Jamain, Emanuele Boselli, Tristan Richard, Josep Valls Fonayet

**Affiliations:** 1 UMR 1366 OENO, Institut des Sciences de la Vigne et du Vin (ISVV), Bordeaux Sciences Agro, Bordeaux INP, University of Bordeaux, INRAE, 33140 Villenave d’Ornon, France; 2 Faculty of Science and Technology, Free University of Bozen-Bolzano, Piazza Università 5, 39100 Bolzano, Italy; emanuele.boselli@unibz.it; 3 Oenolab, NOI Techpark Alto Adige/Südtirol, Via A. Volta 13A, 39100 Bolzano, Italy; 4 Bordeaux Metabolome, MetaboHUB, PHENOME-EMPHASIS, 33140 Villenave d’Ornon, France

**Keywords:** metabolomics, HRMS, conservation, stability, kinetics, flavonoids, stilbenes, cultivar discrimination, Merlot, Cabernet

## Abstract

Metabolomic approaches for wine analysis provide multiple data points for determining quality and traceability. However, sample storage is a major bottleneck and can strongly affect wine composition. We aliquoted wine samples into small tubes and stored them under 4 different conditions (room temperature, +4 °C, −20 °C, and −80 °C). We tracked wine polyphenol composition over two years using targeted HPLC-HRMS. We established the need for reliable quality controls kept at −80 °C to correct batch effects during the study. We observed a general stability trend: phenolic acids > flavonols > flavanols > stilbenes. However, each class showed specific behaviours. As expected, −20 °C and −80 °C were the best conditions and were nearly equivalent, while room temperature caused rapid degradation of almost all polyphenols. Interestingly, we could find satisfactory stability for most polyphenols at +4 °C after 6 months. This was confirmed by assessing the ability to discriminate wines by cultivar. After 24 months of storage, we observed no discrimination for samples kept at room temperature. Our results improve the understanding of how wine storage affects metabolomic studies when samples cannot be collected and analysed simultaneously.

## 1. Introduction

The development of UHPLC-MS methodologies in metabolomics is enabling faster (high-throughput) and more extensive (high-density) coverage of wine composition [1]. Much attention has been given to the analysis of wine polyphenols, which include different closely related families: flavonoids, including anthocyanins, flavan-3-ols, and flavonols, and non-flavonoids, including phenolic acids derived from hydroxycinnamic and hydroxybenzoic acids, and stilbenes such as resveratrol [2]. These compounds play a major role in wine quality because they determine many organoleptic properties, including colour, tannic structure, astringency, bitterness, and ageing potential [3].

The selectivity of UHPLC-MS has enabled faster analysis of multiple polyphenols in short periods of time to study wine properties and quality, authentication, and varietal and geographical traceability [4,5,6,7]. As a consequence, it has become common to analyse large cohorts of wine samples for metabolomics studies [8]. These studies commonly assess the reliability of analytical HPLC-MS methods, but this is not the only critical step in metabolomic experimental design. Sample stability is crucial to ensure the scientific relevance of these studies [8]. The time lag between sample collection and actual analysis, as well as the conditions under which samples are stored, are factors that influence the chemical composition—a seemingly logistical issue but one of paramount analytical importance, as the measured metabolomic profile may no longer accurately reflect the state of the wine at the time of sampling. However, systematic studies on the effects of sample storage conditions on metabolomics measurements remain limited [9].

Wine polyphenols are highly prone to these metabolomic profile changes. Wine is indeed a particularly complex and constantly evolving matrix [10], whose chemical composition results from dynamic equilibria that persist well after bottling: redox reactions, tannin polymerisation/condensation, hydrolysis, and residual enzymatic or microbial activity [11,12,13,14]. These phenomena are highly sensitive to storage conditions (temperature, light, container type), so any analytical measurement depends heavily on the precise moment it is taken [15,16]. Issues relating to the overall sensory and chemical evolution of bottled wine over the long term, considering different conservation parameters (temperature, light, type of closure, bottle position), have been the subject of a relatively extensive body of research from an oenological and sensory perspective, over time scales ranging from the order of a month to several years [16,17,18]. Conversely, few, if any, specific references address the stability of wine samples intended for metabolomic MS analysis in ways relevant to routine analytical practice. This gap is more problematic given that experimental protocols in wine metabolomics implicitly rely on the—rarely verified—assumption of sufficient sample stability between sampling and analysis.

Against this backdrop, the present study aims to assess wine sample stability over time. Our main objective was to characterise potential biases introduced by pre-analytical storage conditions in the metabolomic profile measured by HRMS and to propose methodological recommendations to enhance the reliability of future metabolomic studies applied to wine.

## 2. Results and Discussion

### 2.1. Experimental Design

**Figure 1:**
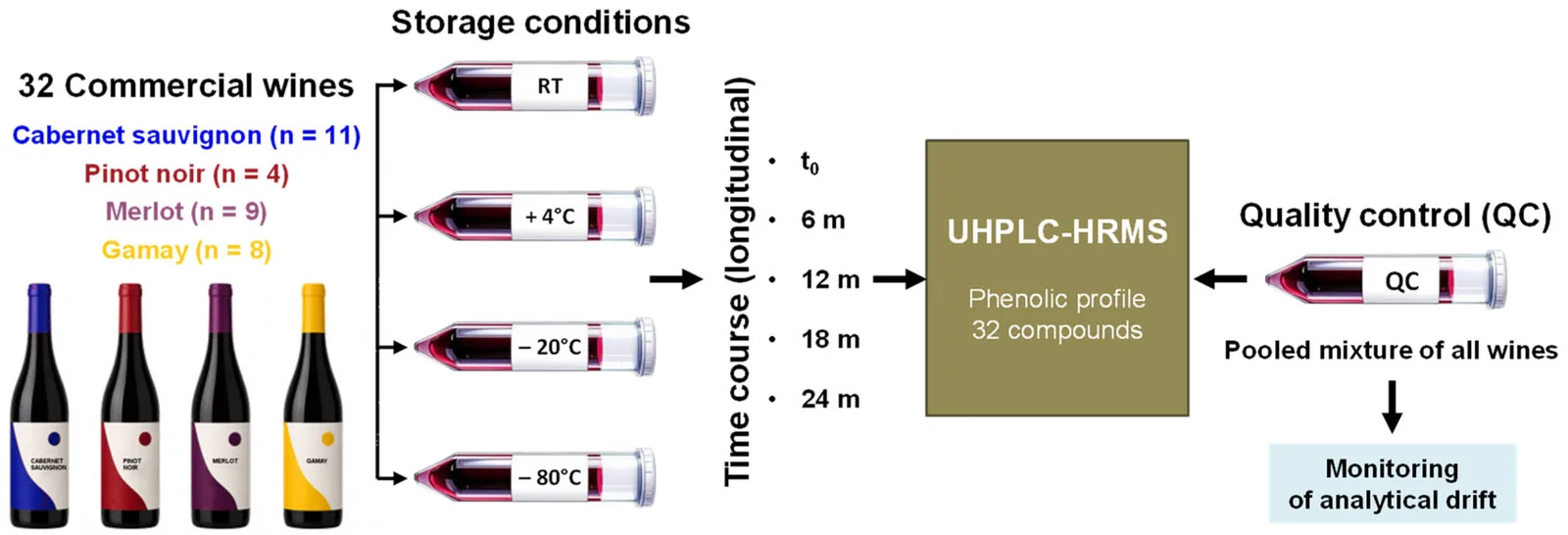
Experimental design.

In this study, 32 monovarietal wines were analysed by HPLC-MS to assess the effect of storage conditions on the stability of phenolic compound signatures. The experimental workflow is illustrated in Figure 1.

Approximately 2 mL of each wine was sampled from the original bottle and transferred into Eppendorf tubes. Sufficient replicates were prepared to cover all storage conditions and analytical time points. Aliquots were then labelled individually and stored at four temperatures: room temperature (RT), +4 °C, −20 °C, and −80 °C. In addition, a pooled quality control (QC) sample was prepared by combining equal volumes of all wine samples. QC aliquots were processed in the same manner as study samples and stored under identical conditions.

Analyses were conducted at 6, 12, 18, and 24 months of storage to assess temporal changes. A total of 32 phenolic compounds were analysed, including phenolic acids, stilbenes, and flavonoids. Table 1 lists the compounds analysed and includes quantification of the pooled QC sample at the initial time. This initial QC composition, representative of the overall wine composition, was used to evaluate potential shifts across batch analyses and sample stability over time at different storage temperatures.

### 2.2. Stability of Pooled QC Samples

**Figure 2:**
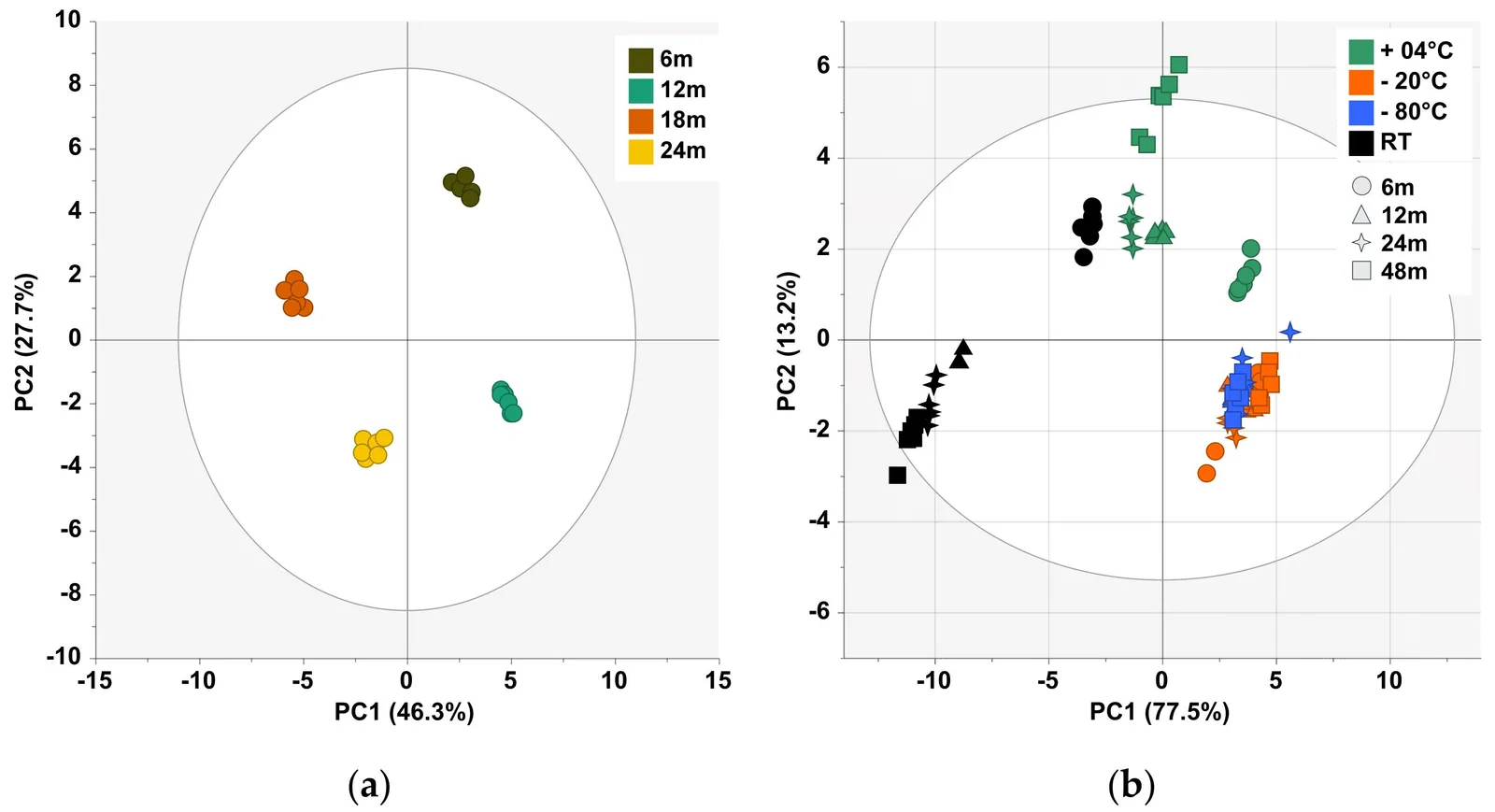
Stability assessment of QC samples. (**a**) PCA of QC samples after 6, 12, 18, and 24 months of storage at −80 °C; (**b**) PCA of QC samples stored at room temperature (RT), +4 °C, −20 °C, and −80 °C, evaluated after 6, 12, 18, and 24 months of storage. Data were normalised to the samples stored at −80 °C within each time point (batch). PCA: principal component analysis; m: months. Ellipses represent the 95% confidence interval.

The stability of the analytical system was assessed using quality control samples (QCs). The principal component analysis (PCA) derived from the quantification of phenolic compounds in QCs stored at −80 °C and analysed at different storage times (6, 12, 18, and 24 months) reveals a marked drift between each set of measurements taken at the various storage times (Figure 2a), indicating a batch effect. This observation suggests a drift in the analytical response between different time points. However, the QCs analysed for each batch are grouped together, and the batches are distributed without any specific structuring, suggesting that the observed variability is primarily due to the instrumental response. Batch effects are well described in LC/MS metabolomics [19], and correction by QCs is the most common approach for solving this issue [20]. Figure 2a highlights this effect and indicates the need to develop reference materials for regular use during batch analyses to minimise sources of variance [21]. In our approach, the QCs kept at different temperatures were systematically injected during each batch (0, 6 m, 12 m and 24 m) to ensure this correction. However, because the QC samples may also evolve over long periods, we used the QC kept at −80 °C as our reference material. This choice is also justified by common sample-conservation protocols for metabolomic studies, which consider −80 °C the optimal storage condition to minimise shifts in metabolite content [9]. However, this approach may not be effective for all wine molecules, especially organic acids such as tartaric acid, which can precipitate irreversibly during freezing.

By normalising data at each time point to the QCs stored at −80 °C, batch effects were reduced, and this enabled direct comparison between storage temperatures, as shown in Figure 2b. This figure shows a second PCA performed on all QCs from the different temperature conditions and analysis times, normalised to the −80 °C QC.

A clear structure by storage temperature is observed, suggesting that storage temperature induces changes in the analytical profiles, independent of instrumental variability. Samples stored at RT and +4 °C diverge markedly from those stored at −80 °C as early as 6 months. Samples stored at −20 °C appear more stable but also exhibit divergence at the longest storage times.

The results indicate that both storage duration and temperature affect the stability of wine polyphenols and confirm a significant temporal drift that must be accounted for when comparing datasets acquired at different time points.

### 2.3. Compound-Dependent Stability

**Figure 3:**
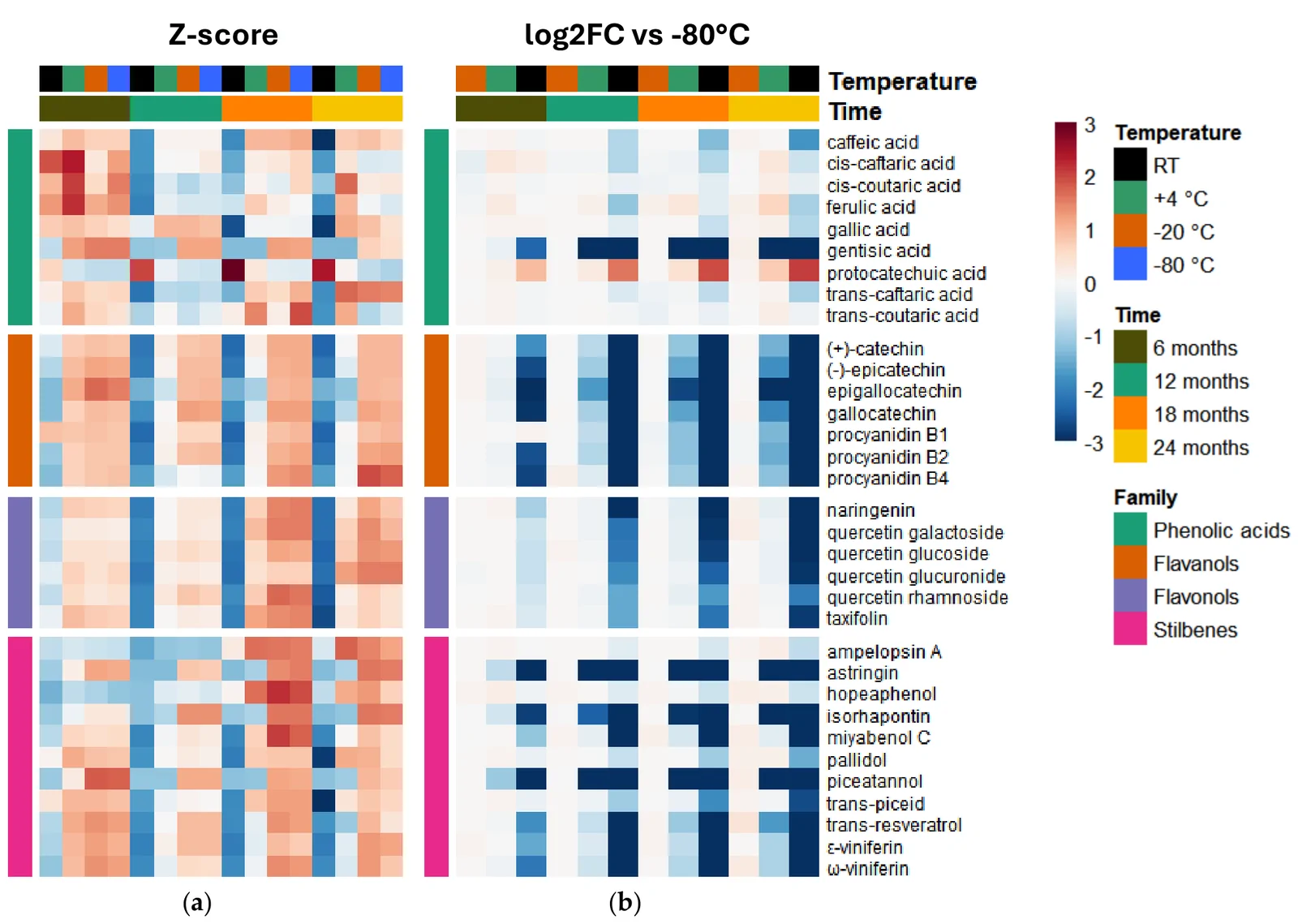
Storage effects on wine polyphenol profile. (**a**) Heatmap of Z-scores derived from log10-transformed concentrations of each polyphenolic compound across storage conditions. (**b**) Heatmap of log2 fold changes relative to the −80 °C condition at each time point. RT: room temperature.

To further study the effect of storage conditions on the chemical composition of wine samples, a detailed monitoring of the contents of each phenolic compound was performed using the QC samples. The results are presented in the form of a heatmap, based on standardised Z-scores (Figure 3a), organised by the 4 different storage temperatures (RT, +4 °C, −20 °C and −80 °C), and sub-organised by the four kinetic points considered (6, 12, 18 and 24 months). The heatmap clearly shows underexpression of most phenolic compounds at RT, evident after 6 months and much more pronounced after 12 months of storage. The +4 °C storage also shows a gradual underexpression of phenolic compounds, but these effects are still moderated after 6 months. Expression under −20 °C and −80 °C storage remains similar even after 24 months of storage. Figure 3b reinforces these results by showing the Log2 fold-change analysis of the RT, +4 °C, and −20 °C relative to −80 °C, the reference condition. This figure confirms the initial impression and provides a concrete assessment of deviations for each compound under the storage conditions.

**Figure 4:**
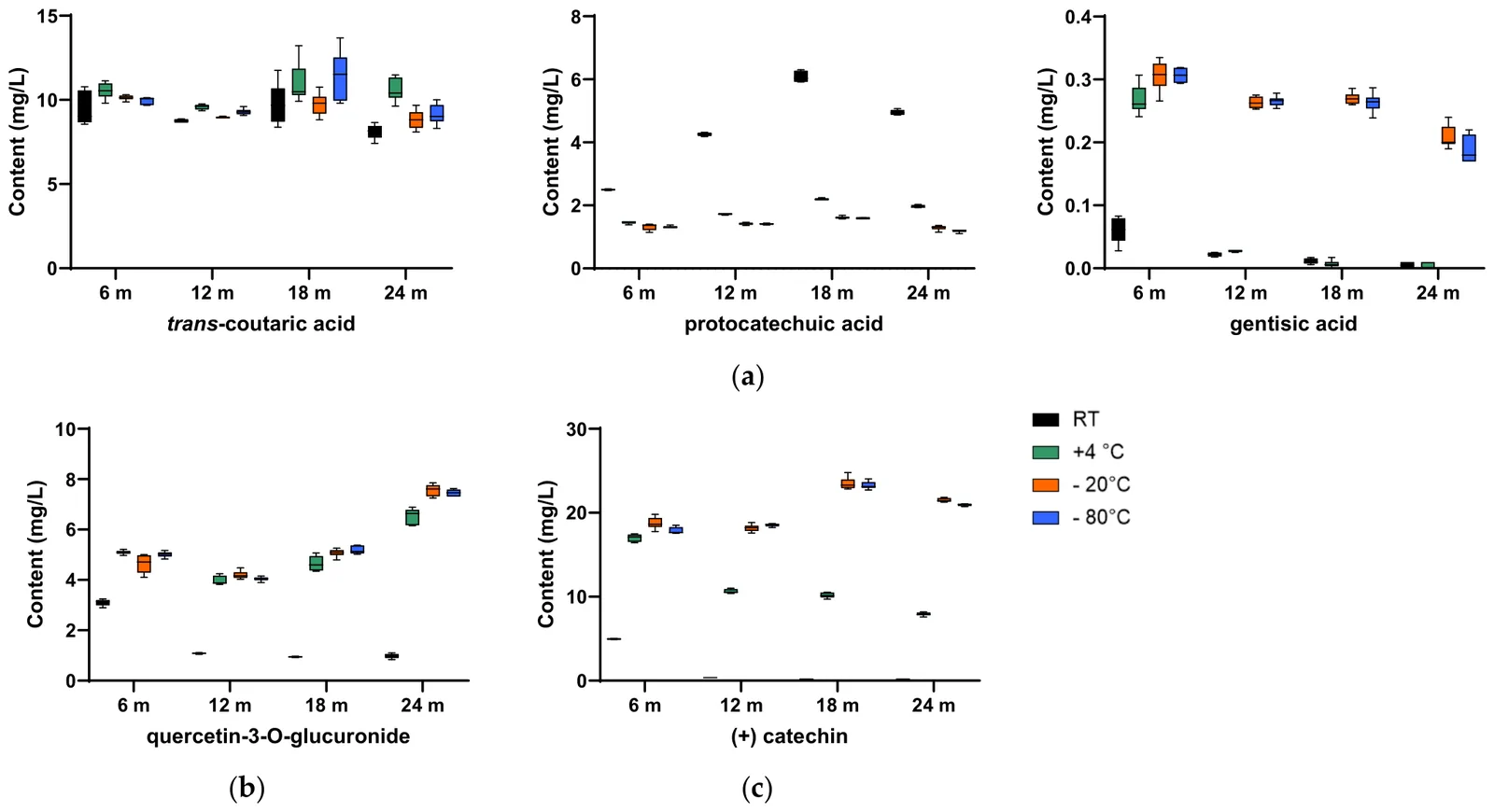

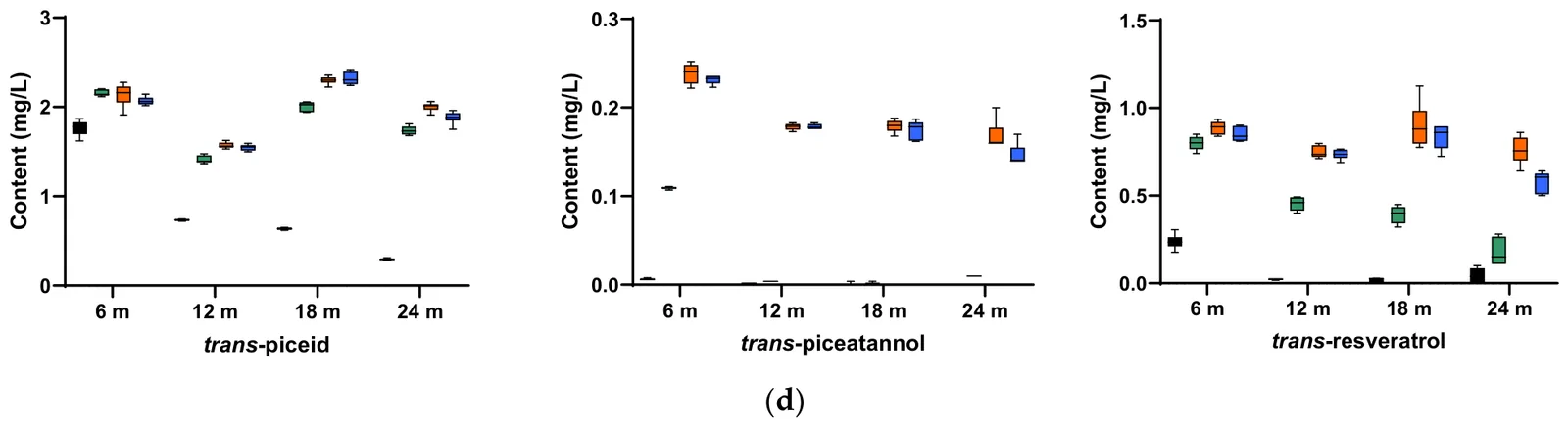
Evolution of phenolic compound concentrations (mg·L^−1^) during storage (6 to 24 months) at room temperature (RT), +4 °C, −20 °C and −80 °C. (**a**) *trans*-coutaric acid, protocatechuic acid and gentisic acid; (**b**) flavonol: quercetin-3-O-glucoside; (**c**) flavanol: (+)-catechin; (**d**) stilbenes: *trans*-piceid, *trans*-piceatannol and *trans*-resveratrol. Values are expressed as mean ± standard deviation Each QC (RT, +4 °C, −20 °C and −80 °C) was injected in sixtupllicate (*n* = 6 for each time of study. m: months.

Beyond the general trend, the heatmap also reveals marked heterogeneity in responses across polyphenol families and even within compound classes, indicating that polyphenols within the same class can exhibit markedly contrasting stability profiles during storage. To complement the multivariate heatmap analyses and illustrate each compound’s stability under different storage conditions, Figure 4 presents boxplots for representative compounds from each family. Boxplots for all compounds are provided in the Supporting Information (Figure S1).

Among the phenolic compound classes studied, Phenolic acids (Figure 4a and Figure S1) generally exhibited the highest stability over time, regardless of storage temperature. This observation likely reflects their relatively simple structure and lower susceptibility to oxidation. Flavonols also showed relatively stable profiles, except at RT, as shown by the boxplot of quercetin glucuronide (Figure 4b and Figure S1). In contrast, flavanols and stilbenes are more sensitive to storage conditions (Figure 4c,d and Figure S1).

The higher chemical stability of phenolic acids than flavanols has been documented in other agrifood matrices [22,23], even over short periods (24 h) under extreme pH conditions [22]. In berry sample storage, the same trend of stability of phenolic acids > flavonols > flavanols was also observed [23]. Giovanelli and Brenna [24] compared the stability of wines stored in plastic bottles, similar to our storage, with different oxygen permeability, over 6 months at 20 °C and 30 °C. They also observed minor changes in phenolic acids such as gallic acid, caftaric acid and coutaric acid, while a marked decrease in flavanols catechin, epicatechin and procyanidin B2 was found. Galmarini et al. [25] also studied the stability of polyphenols on a maltodextrin dried wine powder, over 70 days at 28 °C and 38 °C, showing also that phenolic acids (caftaric, caffeic, gallic acids) as well as quercetin 3-glucoside and resveratrol remained almost constant during 70 days of storage, while flavanols such as catechin, epicatechin or epicatechin gallate, showed slight (about 15–25%) or strong losses (61% loss for epigallocatechin).

Our sample storage conditions were intended to reflect common procedures in metabolomic studies, where a large cohort of samples must be kept before analysis. This choice likely introduces bias relative to the natural evolution of wine in the bottle. This explains the different conservation data for polyphenols in wine bottles reported in other studies. For example, Guo et al. [26] did not observe significant decreases in flavanols during 15 months of storage at RT. However, they also observed better conservation of these compounds at low temperatures (in a cave at 13 °C). However, other research has documented a decline in flavanols during bottle storage after 6 months. Zhang et al. [27] found only a 28% loss of catechin and epicatechin during the first 3 months of dark storage at RT.

While the stability trend phenolic acids > flavonols > flavanols > stilbenes was general, heterogeneity within each phenolic family is evident. Therefore, the chemical class of a compound is not sufficient to predict the long-term behaviour of a phenolic compound under different storage conditions.

For example, in the case of phenolic acids, *trans*-coutaric acid exhibits a stable profile over time regardless of the storage temperature; gallic acid and caffeic acid are also stable except at RT; gentisic acid tends to decrease at RT after 6 months and at +4 °C after 12 months, while protocatechuic acid tends to increase in concentration at RT (Figure 4a and Figure S1). Protocatechuic acid may arise from the degradation of other phenolic compounds, mainly anthocyanins, by scission of the B-ring portion of the cyanidin derivatives [28]. Gallic acid could also be generated from delphinidin derivatives, while syringic acid and vanillic acid could come from peonidin and malvidin, respectively [29,30]. However, these compounds were not determined in our study. Anthocyanins usually require specific methodologies with highly acidified solvents, which limits their use with HPLC-HRMS instrumentation [31]. Moreover, the reactivity of anthocyanins and their formation of other compounds during wine storage has been well documented elsewhere [32,33].

The variability in response is also shown by the behaviour of stilbenes (Figure 4d). Compounds such as piceid, piceatannol, and resveratrol, which share a common chemical structure, exhibit different stability profiles. Monomeric stilbenes (resveratrol and piceatannol) were stable only when frozen (−20 °C and −80 °C) and degraded rapidly at RT. Resveratrol remained stable at +4 °C for 6 months but was completely degraded after 12 months. Piceatannol degradation was even more pronounced, occurring as early as 6 months at +4 °C. In contrast, *trans*-piceid (a glucosylated form of trans-resveratrol) showed better stability than resveratrol, remaining well conserved at +4 °C compared with the most stable conditions. This suggests that conjugation with a sugar can mitigate polyphenol reactivity. This may also explain the stability observed for the glycosylated flavonols (Figure 4c and Figure S1), for which +4 °C may be sufficient even after 24 months of storage. Stilbene dimeric forms (such as ε-viniferin, ω-viniferin, or pallidol) showed better stability compared to the monomeric forms. Although they also degraded at RT, they decreased by a much smaller proportion at +4 °C than the monomers. Interestingly, ampelopsin A and pallidol showed greater stability than most stilbenes. Their structures may explain this: stilbenes often include a conjugated double bond that promotes isomerisation and oxidation reactions [34], whereas ampelopsin A and pallidol lack this double bond. This observation supports the hypothesis that compound stability during storage depends strongly on subtle structural differences (e.g., glycosylation, conjugation, or degree of polymerisation).

Flavanols showed the most homogeneous results, with rapid disappearance at RT after 6 months and at +4 °C after 12 months for all molecule types. However, dimerisation may provide some protection for Procyanidin B1 for up to 6 months at RT and for B4 for up to 12 months at +4 °C. Flavanols are more sensitive to storage conditions, likely because oxidation mechanisms involve quinones [11,35].

### 2.4. Persistence of Wine-Specific Phenolic Signatures During Storage

**Figure 5:**
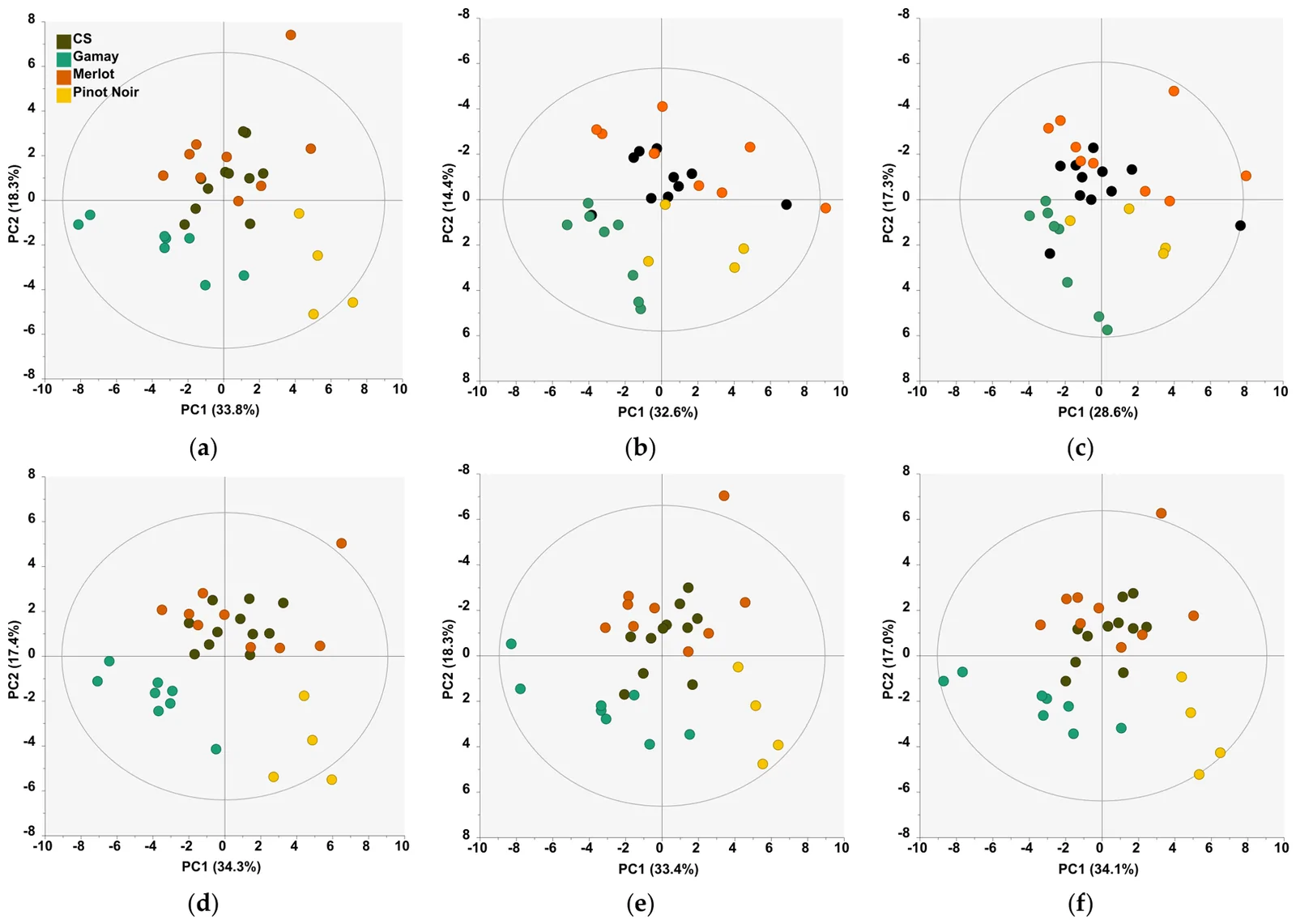
Principal component analysis (PCA) of monovarietal wine samples. (**a**) Initial time; (**b**,**c**) after 12 and 24 months at RT; (**d**–**f**) after 24 months at +4 °C, −20 °C and −80 °C. PCA: principal component analysis; CS: cabernet sauvignon. Ellipses represent the 95% confidence interval.

In this study, 32 monovarietal wines of three cultivars (Cabernet Sauvignon, Merlot, Gamay and Pinot Noir) were analysed (Table S1). A Principal Component Analysis (PCA) was used to assess how storage conditions affect the ability to discriminate these wines (Figure 5). Despite the temperature-induced modifications to the phenolic profiles of the wines, the wine samples remained grouped by variety even after 24 months of storage, except for wines stored at RT, where the separation by grape variety disappeared after 12 months (Figure 5b). These results show that the intrinsic differences in phenolic profiles are stronger than those induced by storage conditions.

Gamay and Pinot Noir wines appear on the negative side of PC2, with Pinot Noir on the positive side of PC1 and Gamay on the negative side. Merlot wines are on the positive side of PC2. Finally, Cabernet Sauvignon wines show a more central distribution. Even if some sample dispersion can be observed depending on storage conditions, the structuring of samples by grape variety remains apparent, even after 24 months.

These results show that each wine has a specific phenolic signature that is preserved over time, even when stored at +4 °C. The phenolic signature of wines can be linked to varietal differences that may induce varying levels of phenolic compounds, flavonols, and stilbenes [1]. The observation of these differences over time, even in wines stored at +4 °C for 24 months, demonstrates that storage conditions more strongly affect the individual concentrations of each phenolic compound than the relative proportions that enable wines to be structured by grape variety in PCA. The ability to preserve varietal classification after low-temperature storage is remarkable, particularly at +4 °C, where the phenolic profile changes significantly. Although the degradation process affects all compounds, the varietal signature remains preserved even after 24 months of storage.

## 3. Materials and Methods

### 3.1. Wine Samples

Thirty-two commercial red wines from the Pays d’Oc region (France) were previously collected from local supermarkets (Table S1, Supplementary Material). The wine set included different grape varieties (Merlot, Cabernet Sauvignon, Gamay) and vintages. Detailed information on grape varieties, vintages, and alcohol content is provided in Table S1.

### 3.2. Sample Preparation and Storage Conditions

Approximately 2 mL of wine was sampled from each bottle using a Coravin© wine preservation system and transferred to Eppendorf^®^ tubes (Montesson, Île-de-France, France). For each wine, a total of 16 tubes were prepared to avoid reusing an old tube at each of the four analytical time points. This ensured that frozen samples had not been previously defrozen/refrozen during the experiment.

Each tube was individually labelled, placed in a dedicated storage box, and stored under one of the following conditions: RT (25 °C) (in a controlled-temperature room), +4 °C, −20 °C, or −80 °C. Samples were analysed after 6, 12, 18, and 24 months of storage after centrifugation (5 min, 10 000 rpm, +4 °C). Quality control samples (QCs) were prepared by mixing equal volumes of all wine samples. They were stored and analysed in the same way as wine samples.

### 3.3. UHPLC-HRMS Analysis

Samples were analysed using a Vanquish UHPLC system (Thermo Fisher Scientific, Bremen, Germany) coupled to a Q-Exactive Plus mass spectrometer (Thermo Fisher Scientific, Bremen, Germany). A volume of 1 µL was injected onto a Luna^®^ Omega Polar C18 column (50 × 2.1 mm, 1.6 µm particle size) supplied by Phenomenex France (Saint-Germain-en-Laye, France).

HRMS data were collected in negative electrospray ionisation mode, adapting a general UHPLC-HRMS methodology successfully used for targeted [36] and untargeted approaches [37]. Chromatographic separation was performed at 40 °C with a flow rate of 0.5 mL/min using solvent A (milli-Q water containing 0.1% formic acid) and solvent B (acetonitrile LC-MS Optima containing 0.1% formic acid LC-MS Optima). The gradient elution program was as follows: 0–7.5 min, 1–36%; 7.5–8.5 min, 36–95%; 8.5–10 min, 95%; 10–12 min, 1% (solvent B), and using the following source parameters: spray voltage, 3000 V; sheath gas, 45 a.u.; auxiliary gas, 15 a.u; capillary temperature, 320 °C; probe heater, 250 °C; and S-lens RF level: 100. Full MS scans were acquired over a mass range of *m*/*z* 70–1050 *m*/*z* at a resolution of 35,000 FWHM, with an AGC target of 3 × 10^6^ ions and a maximum injection time of 100 ms. For compound annotation, additional data-dependent MS/MS spectra were acquired for the five most intense precursors using normalised collision energies of 15, 30, and 40 eV. MS/MS scans were recorded at a resolution of 17,500 FWHM, with an AGC target of 1 × 10^5^ ions and a maximum injection time of 50 ms.

Calibration curves were obtained using 36 polyphenol standards, including flavanols, flavonols, phenolic acids and stilbenes (Table S2, Supplementary Material). Targeted data analysis was performed using Xcalibur software (Thermo Fisher Scientific, version 4.6.67.17). Peaks were extracted from full-scan data based on the exact mass of the deprotonated ion and integrated using the Genesis or Avalon algorithm. Quantification was achieved by comparison with external calibration curves. Because authentic standards for the *cis* isomers are lacking, quantification was performed using the calibration curve for the corresponding *trans* isomers.

### 3.4. Statistical Analysis

Multivariate statistical analyses were used to explore variations in phenolic profiles across storage conditions. Principal component analysis (PCA) was conducted using SIMCA software (Umetrics, version 18, Sartorius France, Aubagne, France) with unit variance (UV) scaling.

Heatmaps were generated in RStudio (version 2026.01.2). Concentrations were log10-transformed [log10(x + 1)] and standardised compound-wise as Z-scores, which were subsequently averaged across replicates for each storage condition. Storage-induced changes were expressed as log2 fold changes (log2FC), calculated for each compound and time point using the mean concentration at −80 °C as the time-matched reference. −80 °C samples were used as reference samples for fold-change calculations. Heatmaps were generated using the pheatmap package. Kinetic data were analysed using GraphPad Prism version 11.0.0 (GraphPad Software, Boston, MA, USA). The effects of storage time and temperature on individual phenolic compound concentrations were assessed by two-way ANOVA, including time, temperature, and their interaction, followed by Tukey’s multiple-comparisons test.

## 4. Conclusions

The results demonstrate the need for a compound-dependent approach to assess the stability of phenolic compounds over time under different storage conditions. Overall, the stability of the studied compounds can be ranked as follows: phenolic acids > flavonols > flavanols > stilbenes.

Our study also provides valuable results for convenient sample storage in wine metabolomic studies, where sampling and analysis often cannot be performed at the same time and where large cohorts may be analysed in different batches. Frozen wine samples are best preserved, with no significant differences between components at 6 and 12 months. However, a very small variation could be found after 18 months of storage for some phenolic acids and stilbenes. Wine samples show significant deviation after just six months of conservation at RT. Interestingly, storage at +4 °C preserves most compounds well for up to 6 months, and this temperature could also suit discrimination studies even after 24 months. From an analytical perspective, although it is preferable to store wine samples frozen, the results suggest that while storage conditions must be carefully controlled when investigating absolute concentrations, multivariate phenolic fingerprints at +4 °C without freezing can remain sufficiently stable to preserve varietal discrimination over extended storage periods. While this study focused on targeted polyphenol analysis, the results could also apply to future untargeted approaches.

## Figures and Tables

**Table 1:** Phenolic compounds quantified in quality control (QC) wine samples. Initial concentrations (t_0_) in QC samples are expressed in mg·L^−1^.

Rt (min)	Compound Name	Concentration (mg·L^−1^)
	*Phenolic acids*	
0.82	gallic acid	27.00 ± 0.57
1.56	gentisic acid	0.30 ± 0.02
1.70	*cis*-caftaric acid	0.25 ± 0.02
2.07	*trans*-caftaric acid	32.47 ± 0.76
2.23	protocatechuic acid	1.49 ± 0.04
2.50	*cis*-coutaric acid	2.91 ± 0.09
2.68	*trans*-coutaric acid	8.66 ± 0.23
3.24	caffeic acid	3.04 ± 0.05
4.55	ferulic acid	0.17 ± 0.04
	*Flavanols and proanthocyanins*	
1.92	gallocatechin	1.94 ± 0.05
2.96	epigallocatechin	0.75 ± 0.03
2.98	procyanidin B1	20.03 ± 0.42
3.05	(+)-catechin	21.94 ± 0.57
3.42	procyanidin B4	0.34 ± 0.03
3.73	procyanidin B2	6.56 ± 0.34
3.77	(−)-epicatechin	12.39 ± 0.32
	*Flavonols and flavones*	
4.72	Taxifolin	0.38 ± 0.01
5.17	quercetin-3-O-galactoside	0.44 ± 0.05
5.19	quercetin-3-O-glucuronide	4.63 ± 0.15
5.24	quercetin-3-O-glucoside	0.44 ± 0.03
5.70	quercetin-3-O-rhamnoside	0.07 ± 0.01
7.21	naringenin	0.07 ± 0.01
	*Stilbenes*	
4.20	*trans*-astringin	0.32 ± 0.02
4.87	*trans*-piceid	2.18 ± 0.09
5.14	ampelopsin A	0.48 ± 0.03
5.17	*trans*-piceatannol	0.11 ± 0.01
5.93	pallidol	0.48 ± 0.03
6.02	*trans*-resveratrol	1.28 ± 0.28
6.98	hopeaphenol	0.02 ± 0.01
7.65	ε-viniferin	0.09 ± 0.02
7.90	miyabenol C	0.03 ± 0.01
8.09	ω-viniferin	0.05 ± 0.01

## Data Availability

Data are available on request.

## Supplementary Materials

The following supporting information can be downloaded at: https://www.mdpi.com/article/10.3390/molecules31213867/s1, Table S1: List of the 32 purchased wines; Table S2: Polyphenol standards used for targeted UHPLC-HRMS analysis and calibration; Table S3: Calibration parameters for the UHPLC-HRMS methodology of polyphenols; Figure S1: Evolution of phenolic compound concentrations during storage.

## Abbreviations

The following abbreviations are used in this manuscript:

AGC	Automatic Gain Control
CS	Cabernet Sauvignon
FWHM	Full Width at Half Maximum
HPLC	High-Performance Liquid Chromatography
HRMS	High-Resolution Mass Spectrometry
PCA	Principal Component Analysis
QC	quality control
RT	Room Temperature

## References

[1] Valls-Fonayet J., Loupit G., Richard T., Petriacq P., Bouchereau A. (2021). MS- and NMR-metabolomic tools for the discrimination of wines: Applications for authenticity. Plant Metabolomics in Full Swing.

[2] Flamini R. (2003). Mass spectrometry in grape and wine chemistry. Part I: Polyphenols. Mass Spectrom. Rev..

[3] Gutiérrez-Escobar R., Aliaño-González M.J., Cantos-Villar E. (2021). Wine polyphenol content and its influence on wine quality and properties: A review. Molecules.

[4] Ciucure C.-T., Miricioiu M.G., Geana E.-I. (2025). Discrimination of romanian wines based on phenolic composition and identification of potential phenolic biomarkers for wine authenticity and traceability. Beverages.

[5] Martelo-Vidal M.J., Vázquez M. (2016). Polyphenolic profile of red wines for the discrimination of controlled designation of origin. Food Anal. Methods.

[6] Zhang L., Wang Z., Zhang C., Zhou S., Yuan C. (2024). Metabolomics analysis based on UHPLC-QqQ-MS/MS to discriminate grapes and wines from different geographical origins and climatological characteristics. Food Chem. X.

[7] Leleu G., Butelle R., de Moura P.H.B., Valls-Fonayet J., Da Costa G., Richard T. (2026). Wine authenticity controls: Analytical methods, data Integration and emerging perspectives. Food Anal. Methods.

[8] Lukić I., Radeka S., Budić-Leto I., Bubola M., Vrhovsek U. (2019). Targeted UPLC-QqQ-MS/MS profiling of phenolic compounds for differentiation of monovarietal wines and corroboration of particular varietal typicity concepts. Food Chem..

[9] Le Bris N., Beringue A., Rivas J., Renault D., Chorin M., Colinet H. (2025). How to process samples for metabolomics in ecology: A comparative study of preanalytical storage methods. J. Sep. Sci..

[10] Le Mao I., Da Costa G., Richard T. (2022). 1H-NMR metabolomics for wine screening and analysis. OENO One.

[11] Oliveira C.M., Ferreira A.C.S., de Freitas V., Silva A.M. (2011). Oxidation mechanisms occurring in wines. Food Res. Int..

[12] Ailer Š., Jakabová S., Benešová L., Ivanova-Petropulos V. (2022). Wine faults: State of knowledge in reductive aromas, oxidation and atypical aging, prevention, and correction methods. Molecules.

[13] Li H., Guo A., Wang H. (2008). Mechanisms of oxidative browning of wine. Food Chem..

[14] Comitini F., Agarbati A., Canonico L., Ciani M. (2021). Yeast interactions and molecular mechanisms in wine fermentation: A comprehensive review. Int. J. Mol. Sci..

[15] Razafindrabenja L.E., Caillé S., Verbaere A., Meudec E., Galy N., Tixador D., Loisel C., Sommerer N., Mouls L. (2026). Impact of bottle closures on tannin evolution in red wine after 17 years of aging using UHPLC−UHRMS metabolomic approach. J. Food Compos. Anal..

[16] Castro C.C., Martins R.C., Teixeira J.A., Silva Ferreira A.C. (2014). Application of a high-throughput process analytical technology metabolomics pipeline to Port wine forced ageing process. Food Chem..

[17] Garcia L., Martet E., Suc L., Garcia F., Saucier C. (2024). Analysis of targeted phenolic ageing markers in Syrah red wines during bottle ageing: Influence of cork oxygen transfer rate. Food Chem..

[18] Ribéreau-Gayon P., Glories Y., Maujean A., Dubourdieu D. (2006). Handbook of Enology.

[19] Liu Q., Walker D., Uppal K., Liu Z., Ma C., Tran V., Li S., Jones D.P., Yu T. (2020). Addressing the batch effect issue for LC/MS metabolomics data in data preprocessing. Sci. Rep..

[20] Guo F., Lin G., Dong L., Cheng K.-K., Deng L., Xu X., Raftery D., Dong J. (2023). Concordance-based batch effect correction for large-scale metabolomics. Anal. Chem..

[21] Lippa K.A., Aristizabal-Henao J.J., Beger R.D., Bowden J.A., Broeckling C., Beecher C., Clay Davis W., Dunn W.B., Flores R., Goodacre R. (2022). Reference materials for MS-based untargeted metabolomics and lipidomics: A review by the metabolomics quality assurance and quality control consortium (mQACC). Metabolomics.

[22] Pasquet P.L., Julien-David D., Zhao M., Villain-Gambier M., Trébouet D. (2024). Stability and preservation of phenolic compounds and related antioxidant capacity from agro-food matrix: Effect of pH and atmosphere. Food Biosci..

[23] Zhou Y., Saarniit K., Jafari M.S., Rosenvald S., Laaksonen O., Tian Y., Yang B. (2025). Storage stability of berry mueslis with special focus on phenolic compounds. LWT.

[24] Giovanelli G., Brenna O.V. (2007). Oxidative stability of red wine stored in packages with different oxygen permeability. Eur. Food Res. Technol..

[25] Galmarini M.V., Maury C., Mehinagic E., Sanchez V., Baeza R.I., Mignot S., Zamora M.C., Chirife J. (2013). Stability of individual phenolic compounds and antioxidant activity during storage of a red wine powder. Food Bioprocess Technol..

[26] Guo L., Hu Y., Wang D., Li J., Zhang X., Cheng Y., Liu S., Zhang H., Zhao H. (2026). Impact of cave bottle storage on the chemical composition and sensory properties of Marselan wine: Focus on volatiles, phenolics, and color. LWT.

[27] Zhang Y., Zhang Q., Liu J., Nan Y., Cheng X., Fang Y., Sun X., Li J. (2026). Effects of grape seed proanthocyanidins with different polymerization degrees on the phenolic compounds and sensory quality of Cabernet Sauvignon wine during bottle aging. Foods.

[28] Sinela A.M., Mertz C., Achir N., Rawat N., Vidot K., Fulcrand H., Dornier M. (2017). Exploration of reaction mechanisms of anthocyanin degradation in a roselle extract through kinetic studies on formulated model media. Food Chem..

[29] Darnal A., Ceci A.T., Poggesi S., Mimmo T., Boselli E., Longo E. (2026). Chemical, colour, and sensory evolution of Schiava wines: Impact of grape freezing and co-inoculum of yeast and malolactic Bacteria. Food Chem..

[30] Yang M., Zhang M., Wang B., Zhang Z., Zhuang Y., Liu J., Zhang Q., Fei P. (2025). Mechanism-driven stabilization of anthocyanins: Comparative copigmentation and encapsulation for food applications. Food Chem..

[31] Aissat A.K., Chaher-Bazizi N., Richard T., Kilani-Atmani D., Pedrot E., Renouf E., Atmani D., Valls Fonayet J. (2022). Analysis of individual anthocyanins, flavanols, flavonols and other polyphenols in Pistacia lentiscus L. fruits during ripening. J. Food Compos. Anal..

[32] Rentzsch M., Schwarz M., Winterhalter P. (2007). Pyranoanthocyanins—An overview on structures, occurrence, and pathways of formation. Trends Food Sci. Technol..

[33] Gomes V., Mateus N., de Freitas V., Oliveira J., Cruz L. (2023). Pyranoanthocyanins and other pyranoflavylium dyes: From chemistry towards novel applications. Dye. Pigment..

[34] Pébarthé-Courrouilh A., Jaa A., Valls-Fonayet J., Da Costa G., Palos-Pinto A., Richard T., Cluzet S. (2024). UV-exposure decreases antimicrobial activities of a grapevine cane extract against Plasmopara viticola and Botrytis cinerea as a consequence of stilbene modifications-a kinetic study. Pest Manag. Sci..

[35] Bai X., Chen X., Li X., Tan F., Sam F.E., Tao Y. (2024). Wine polyphenol oxidation mechanism and the effects on wine quality: A review. Compr. Rev. Food Sci. Food Saf..

[36] Mairata A., Valls-Fonayet J., Labarga D., Puelles M., Cluzet S., Portu J., Pou A. (2025). Influence of organic mulches and soil properties on the phenolic profile of leaves, canes and grape skins in grapevine (*Vitis vinifera* L.). J. Sci. Food Agric..

[37] Chavonet E., Mirande-Ney C., Bernardo S., Guinand C., Delestre G., Zhan X., Fonayet J.V., Prigent S., van Delft P., Pascal S. (2025). Deciphering heat-enhanced resistance of grapevine berries to Botrytis cinerea highlights differential cuticular and secondary metabolite accumulations between Merlot and Cabernet Sauvignon. Plant Stress.

