# Supplementary material for: Impact of Sample Storage Conditions on Phenolic Profiles of Commercial Wines Assessed by UHPLC-HRMS: Implications for Analytical Reproducibility
Source: Molecules. 2026 Oct 27;31(21):3867. doi: 10.3390/molecules31213867 (PMC13765012; doi:10.3390/molecules31213867)
Supplement: Supplementary file 1 [file molecules-31-03867-s001.zip › molecules-4586412-supplementary.pdf]

Supplementary Material

Impact of Sample Storage Conditions on Phenolic Profiles of Commercial Wines Assessed by UHPLC-HRMS: Implications for Analytical Reproducibility

Nahikari Prevost [1,2,3], Alan Jamain [1], Emanuele Boselli [2,3], Tristan Richard [1,4,*] and Josep Valls Fonayet [1,4,*]

[1] UMR 1366 OENO, Institut des Sciences de la Vigne et du Vin (ISVV), Bordeaux Sciences Agro, Bordeaux INP, University of Bordeaux, INRAE, 33140 Villenave d'Ornon, France
[2] Faculty of Science and Technology, Free University of Bozen-Bolzano, Piazza Università 5, 39100 Bolzano, Italy
[3] Oenolab, NOI Techpark Alto Adige/Südtirol, Via A. Volta 13A, 39100 Bolzano, Italy
[4] Bordeaux Metabolome, MetaboHUB, PHENOME-EMPHASIS, 33140 Villenave d'Ornon, France
* Correspondence: tristan.richard@u-bordeaux.fr (T.R.); josep.valls-fonayet@u-bordeaux.fr (J.V.F.)

Supplementary Material List

Table S1. List of the 32 purchased wines.

Table S2. Polyphenol standards used for targeted UHPLC-HRMS analysis and calibration.

Table S3. Calibration parameters for the UHPLC-HRMS methodology of polyphenols.

Figure S1. Evolution of phenolic compound concentrations during storage.

Table S1. List of the 32 purchased wines.

Sample code	Winery	Alcohol content (%)	Variety	Vintage
CS-08	Pierre Chanau	13.5	Cabernet Sauvignon	2020
CS-09	By Haussmann	13.5	Cabernet Sauvignon	2022
CS-10	Roche Mazet	13	Cabernet Sauvignon	2021
CS-11	Les Ormes de Cambras	13	Cabernet Sauvignon	2021
CS-14	Pierre Chanau	13.5	Cabernet Sauvignon	2021
CS-15	Naturae – Gérard Bertrand	13	Cabernet Sauvignon	2021
CS-16	Autrement – Gérard Bertrand	13.5	Cabernet Sauvignon	2020
CS-17	Floriance	13.5	Cabernet Sauvignon	2021
CS-18	Couleurs du sud	13	Cabernet Sauvignon	2019
CS-19	Les Ormes de Cambras	13	Cabernet Sauvignon	2022
CS-20	Vieux Carion	13	Cabernet Sauvignon	2022
G-04	La Croix du Pin	12	Gamay	2020
G-05	L'Héritage de Carillan	12	Gamay	2019
G-06	Vieux Carion	11.5	Gamay	2016
G-07	Pierre Chanau	12.5	Gamay	2019
G-08	Pierre Chanau	13	Gamay	2020
G-09	Pierre Chanau	12	Gamay	2021
G-11	Infinity – Pierre Jean Larraqué	12	Gamay	2020
G-12	Vieux Carion	12	Gamay	2022
MT-08	Les Ormes de Cambras	13	Merlot	2021
MT-09	Roche Mazet	13	Merlot	2021
MT-12	Naturae Bio	14	Merlot	2020
MT-13	Autrement Bio	13.5	Merlot	2021
MT-14	Impulsive	13	Merlot	2019
MT-15	Grain d'Oc	13.5	Merlot	2017
MT-16	Grain d'Oc	13	Merlot	2015
MT-17	LeVal	13	Merlot	2020
MT-18	Les Ormes de Cambras	13	Merlot	2022
PN-03	Intuitive – Pierre Jean Larraqué	13.5	Pinot Noir	2021
PN-04	Héritage LeVal Occitan	13	Pinot Noir	2022
PN-05	Bonfils – Domaine de Cibadiès	13	Pinot Noir	2021
PN-06	Cœur de Pierre	12.5	Pinot Noir	2021

Table S2. Polyphenol standards used for targeted UHPLC-HRMS analysis and calibration.

RT (min)	Compound	Chemical class	Molecular formula	$[M–H]^-$ (*m/z*)	Standard availability	Quantification approach
0.82	gallic acid	Phenolic acids	$C_7H_6O_5$	169.0142	Commercial standard	External calibration
1.56	gentisic acid	Phenolic acids	$C_7H_6O_4$	153.0193	Commercial standard	External calibration
1.70	*cis*-caftaric acid	Phenolic acids	$C_{13}H_{12}O_9$	311.0408	No commercial standard	Quantified as *trans*-caftaric acid
1.92	gallocatechin	Flavan-3-ols	$C_{15}H_{14}O_7$	305.0666	Commercial standard	External calibration
2.07	*trans*-caftaric acid	Phenolic acids	$C_{13}H_{12}O_9$	311.0408	Commercial standard	External calibration
2.23	protocatechuic acid	Phenolic acids	$C_7H_6O_4$	153.0193	Commercial standard	External calibration
2.50	*cis*-coutaric acid	Phenolic acids	$C_{13}H_{12}O_8$	295.0459	No commercial standard	Quantified as *trans*-coutaric acid
2.68	*trans*-coutaric acid	Phenolic acids	$C_{13}H_{12}O_8$	295.0459	Commercial standard	External calibration
2.96	epigallocatechin	Flavan-3-ols	$C_{15}H_{14}O_7$	305.0666	Commercial standard	External calibration
2.98	procyanidin B1	Flavan-3-ols	$C_{30}H_{26}O_{12}$	577.1351	Commercial standard	External calibration
3.05	(+)-catechin	Flavan-3-ols	$C_{15}H_{14}O_6$	289.0717	Commercial standard	External calibration
3.24	caffeic acid	Phenolic acids	$C_9H_8O_4$	179.0350	Commercial standard	External calibration
3.42	procyanidin B4	Flavan-3-ols	$C_{30}H_{26}O_{12}$	577.1351	No commercial standard	Quantified as procyanidin B2
3.73	procyanidin B2	Flavan-3-ols	$C_{30}H_{26}O_{12}$	577.1351	Commercial standard	External calibration
3.77	(-)-epicatechin	Flavan-3-ols	$C_{15}H_{14}O_6$	289.0717	Commercial standard	External calibration
4.20	*trans*-astringin	Stilbenes	$C_{20}H_{22}O_9$	405.1134	Commercial standard	External calibration
4.55	ferulic acid	Phenolic acids	$C_{10}H_{10}O_4$	193.0506	Commercial standard	External calibration
4.72	taxifolin	Flavonols	$C_{15}H_{12}O_7$	303.0505	Commercial standard	External calibration
4.87	*trans*-piceid	Stilbenes	$C_{20}H_{22}O_8$	389.1242	Commercial standard	External calibration
5.14	ampelopsin A	Stilbenes	$C_{28}H_{22}O_7$	469.1293	Commercial standard	External calibration
5.17	quercetin-3-O-galactoside	Flavonols	$C_{21}H_{20}O_{12}$	463.0882	Commercial standard	External calibration
5.17	*trans*-piceatannol	Stilbenes	$C_{14}H_{12}O_4$	243.0663	Commercial standard	External calibration
5.19	quercetin-3-O-glucuronide	Flavonols	$C_{21}H_{18}O_{13}$	477.0674	Commercial standard	External calibration
5.24	quercetin-3-O-glucoside	Flavonols	$C_{21}H_{20}O_{12}$	463.0882	Commercial standard	External calibration
5.70	quercetin-3-O-rhamnoside	Flavonols	$C_{21}H_{20}O_{11}$	447.0933	Commercial standard	External calibration
5.93	pallidol	Stilbenes	$C_{28}H_{22}O_6$	453.1344	Commercial standard	External calibration
6.02	*trans*-resveratrol	Stilbenes	$C_{14}H_{12}O_3$	227.0714	Commercial standard	External calibration
6.98	hopeaphenol	Stilbenes	$C_{56}H_{42}O_{12}$	905.2597	Commercial standard	External calibration
7.21	naringenin	Flavonols	$C_{15}H_{12}O_5$	271.0606	Commercial standard	External calibration
7.65	ε-viniferin	Stilbenes	$C_{28}H_{22}O_6$	453.1344	Commercial standard	External calibration
7.90	miyabenol C	Stilbenes	$C_{42}H_{32}O_9$	679.1992	Commercial standard	External calibration
8.09	ω-viniferin	Stilbenes	$C_{28}H_{22}O_6$	453.1344	Commercial standard	External calibration

Table S3. Calibration parameters for the UHPLC-HRMS methodology of polyphenols. Intraday precision was measured as the Coefficient of Variation (CV%) of three analyses of a quality control (mixture of all samples) within a day; interday precision was measured as the CV% of three analyses of the quality control on two different days. Accuracy was measured by calculating recovery in a quality control sample spiked with 8 mg/L of each polyphenol. The limits of detection (LOD) were estimated as the lowest concentration on the calibration curve that gave a clear peak distinct from the blank, and the LOQ as 3 LOD. The best fit was a quadratic curve.

	Interval of the curve (mg/L)	equation	R2	CV (%) intraday	CV (%) interday	Recovery (%)	LOD (mg/L)	LOQ (mg/L)
**gallic acid**	0.015-83	Y= 2.11.107 x2 – 107502 x	0.935	0.9%	3.7%	89%	0.005	0.015
**catechin**	0.015-83	Y= 2.48.107 x2 – 122696 x	0.993	1.6%	1.9%	79%	0.005	0.015
**caffeic acid**	0.015-83	Y= 3.45.107 x2 – 179858 x	0.994	17.2%	17.6%	57%	0.005	0.015
**procyanidin b2**	0.015-83	Y= 1.20.107 x2 – 60994 x	0.995	2.3%	3.1%	73%	0.005	0.015
**epicatechin**	0.015-83	Y= 2.88.107 x2 – 161784 x	0.993	1.0%	0.6%	69%	0.005	0.015
**4-coumaric acid**	0.24-83	Y= 2.94.106 x2 – 8670 x	0.999	1.7%	1.5%	67%	0.08	0.24
***trans*-piceid**	0.015-83	Y= 1.06.107 x2 – 52544 x	0.998	3.1%	2.9%	67%	0.005	0.015
**quercetin 3-glucuronide**	0.015-83	Y= 2.67.107 x2 – 151588 x	0.995	0.6%	0.8%	71%	0.005	0.015
**pallidol**	0.015-83	Y= 1.36.107 x2 – 69264x	0.997	1.5%	0.3%	56%	0.005	0.015
***trans*-resveratrol**	0.23-83	Y= 1.06.106 x2 – 1833x	0.999	21.8%	22.9%	60%	0.078	0.227
**hopeaphenol -isomer**	0.06-83	Y= 2.41.107 x2 – 9040 x	0.999	1.8%	0.5%	70%	0.02	0.06
**ε-viniferin**	0.015-83	Y= 2.44.107 x2 – 125920x	0.972	0.4%	0.9%	78%	0.005	0.015

Figure S1. Evolution of phenolic compound concentrations during storage. Statistical significance was assessed using two-way ANOVA followed by Tukey's multiple-comparisons test (*p < 0.033, **p < 0.002, ***p < 0.001).

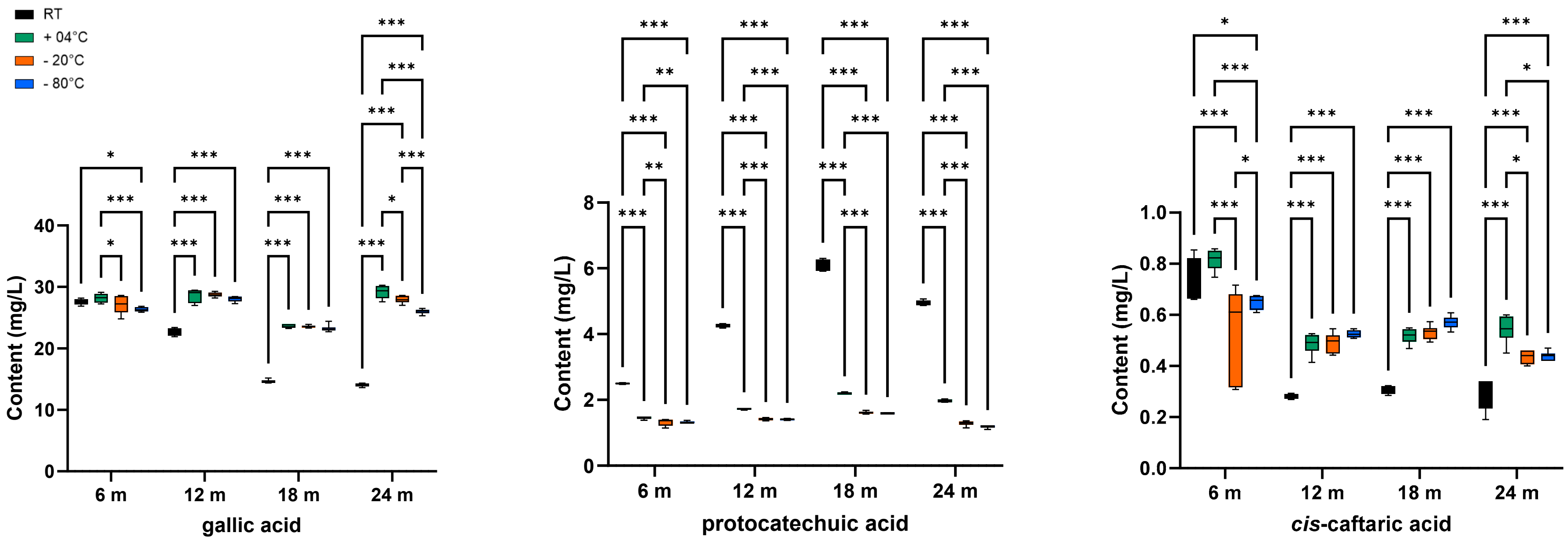

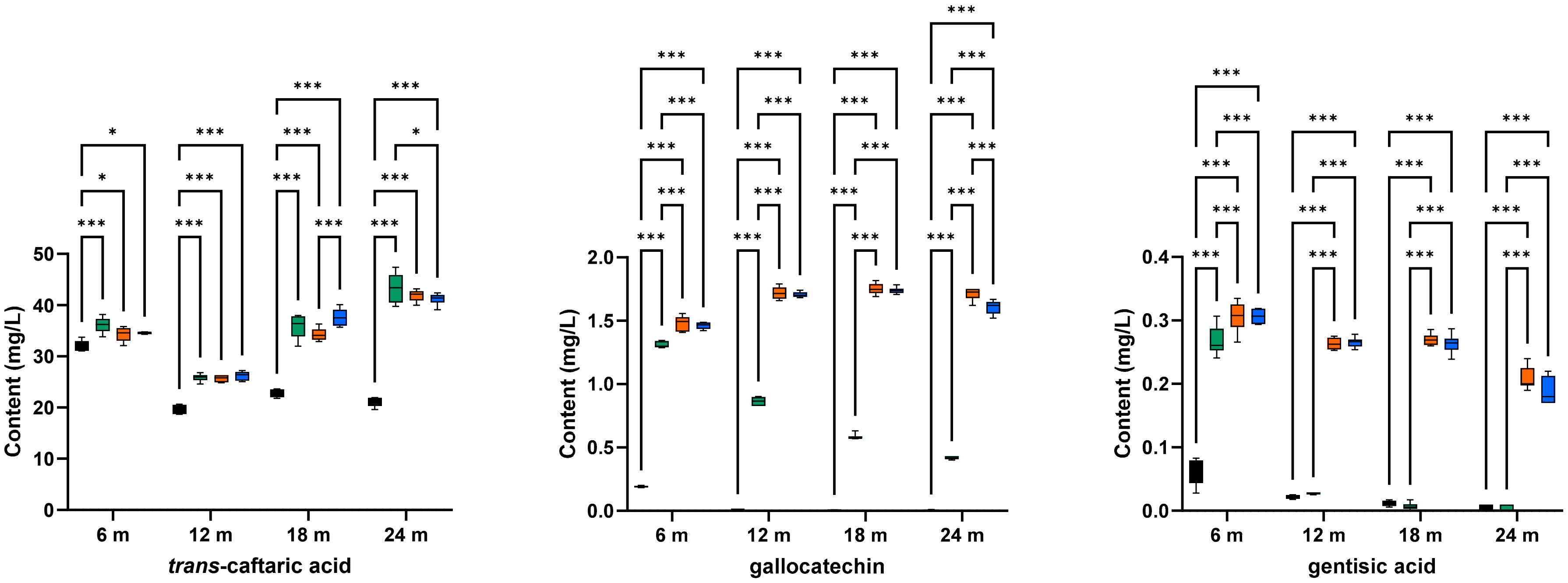
Content (mg/L)
6 m
12 m
18 m
24 m
trans-caftaric acid
gallocatechin
gentisic acid

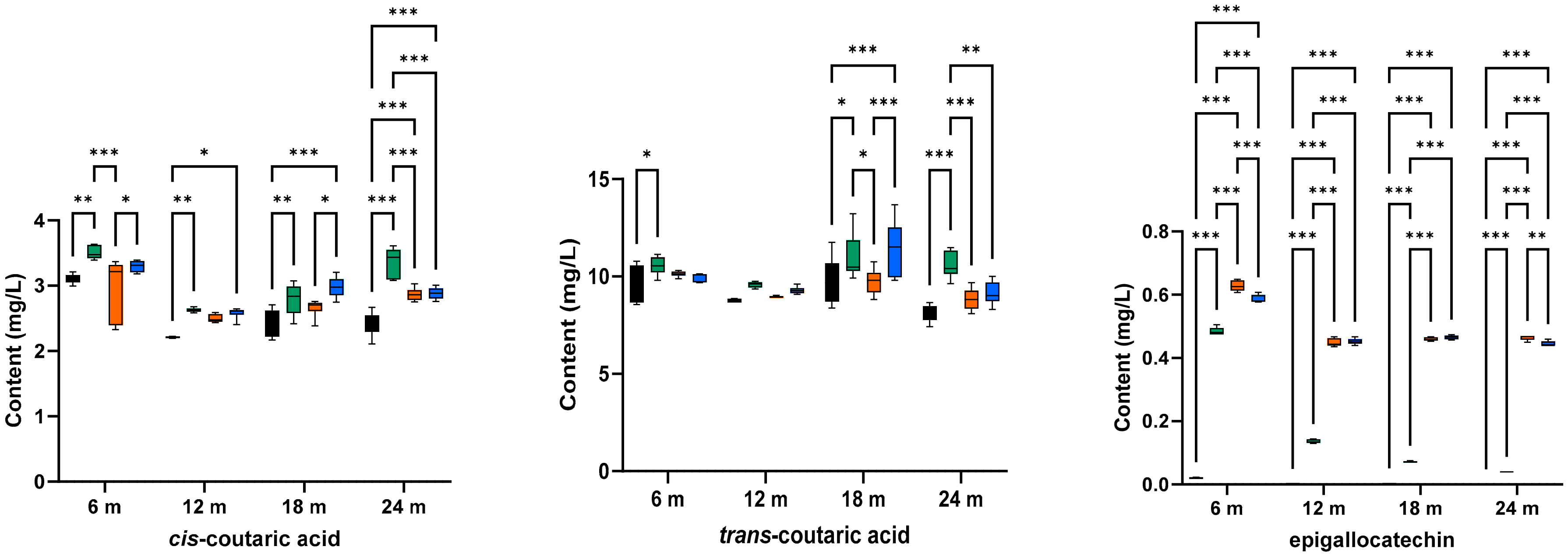

Content (mg/L)
6 m
12 m
18 m
24 m
cis-coutaric acid
trans-coutaric acid
epigallocatechin

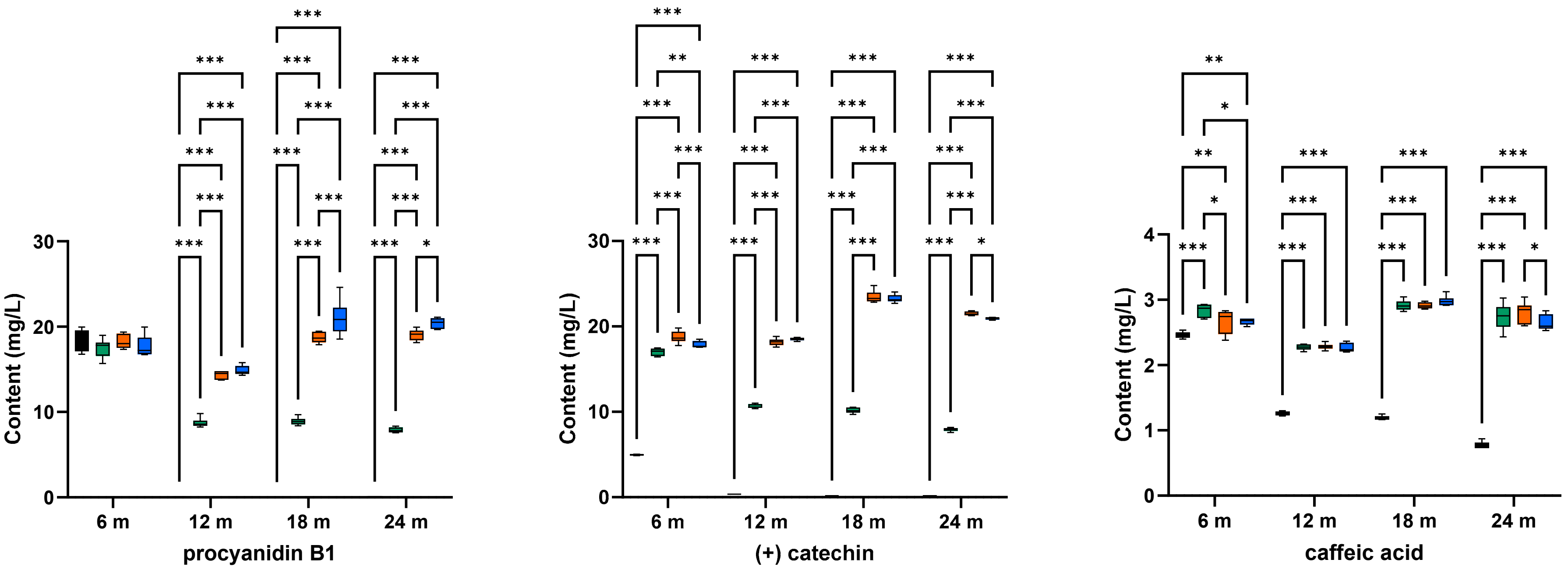
Content (mg/L)
6 m
12 m
18 m
24 m
procyanidin B1
(+) catechin
caffeic acid

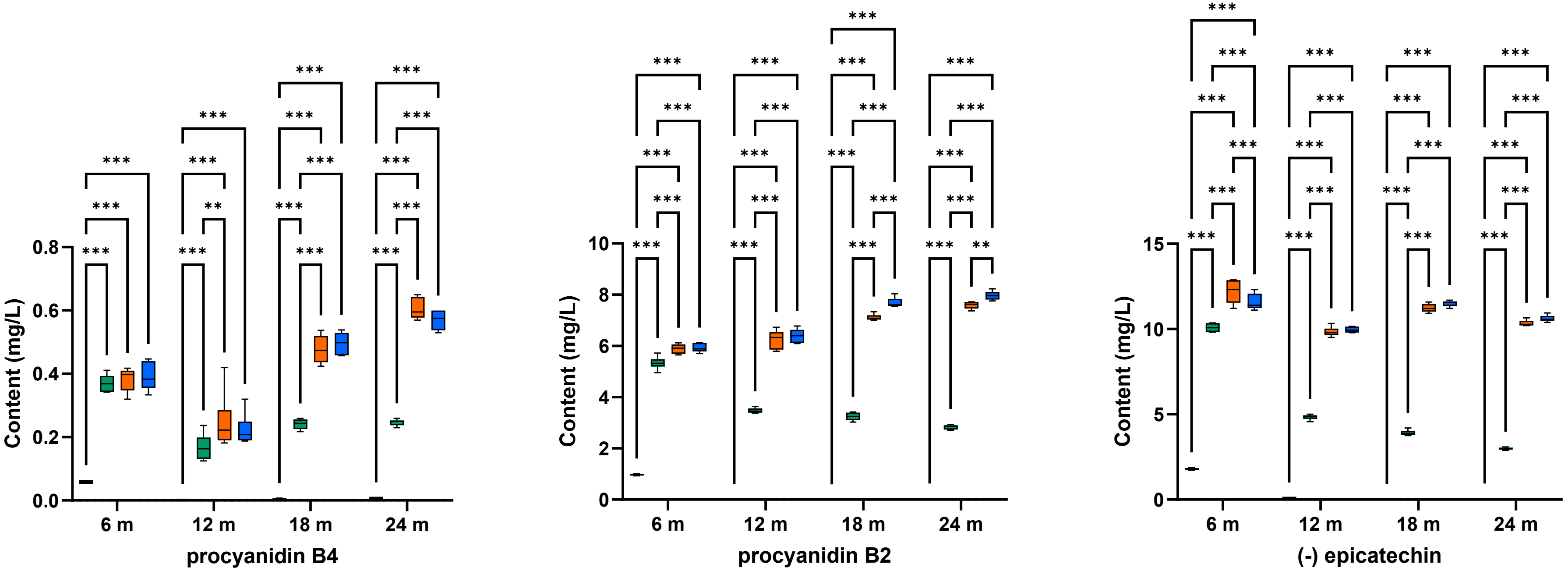
Content (mg/L)
6 m
12 m
18 m
24 m
procyanidin B4
procyanidin B2
(-) epicatechin

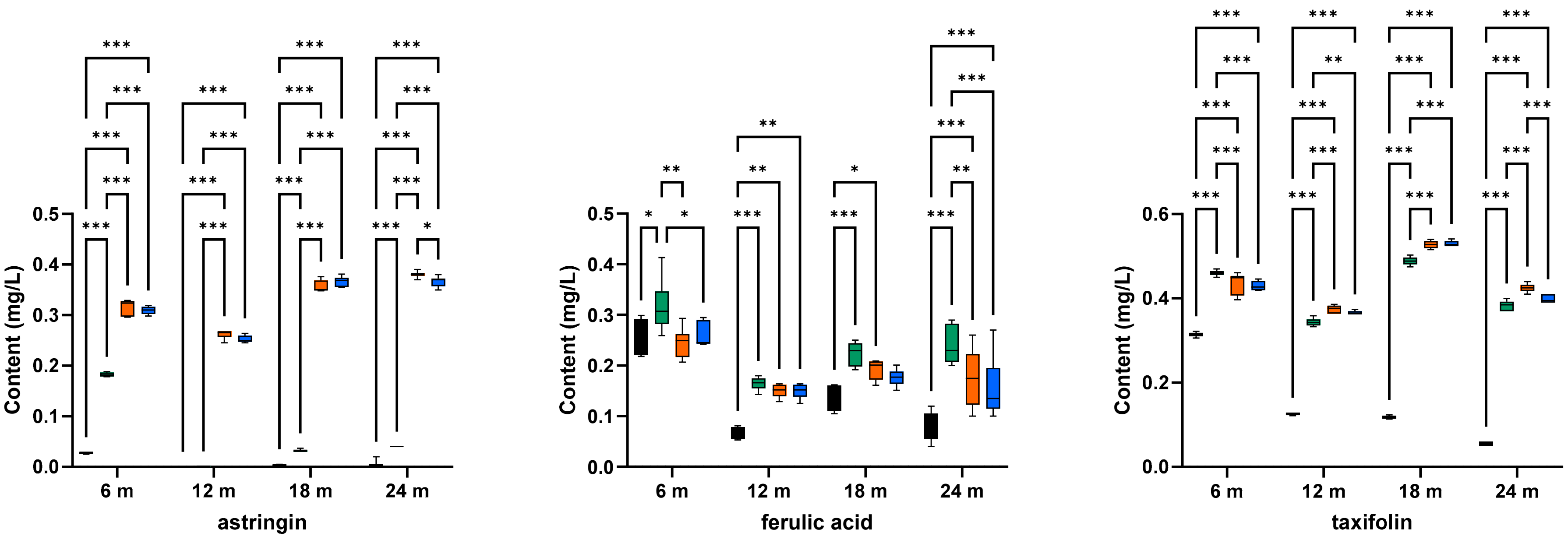
Content (mg/L)
6 m
12 m
18 m
24 m
astringin
Content (mg/L)
6 m
12 m
18 m
24 m
ferulic acid
Content (mg/L)
6 m
12 m
18 m
24 m
taxifolin

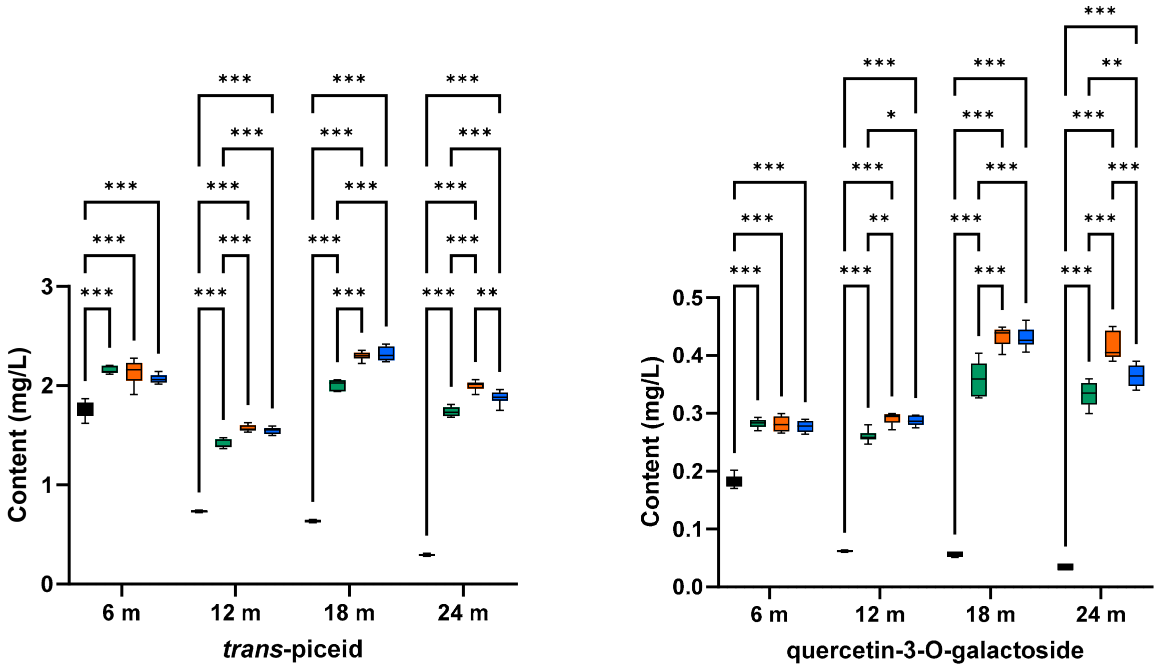
Content (mg/L)
0
1
2
3
6 m
12 m
18 m
24 m
trans-piceid
Content (mg/L)
0.0
0.1
0.2
0.3
0.4
0.5
6 m
12 m
18 m
24 m
quercetin-3-O-galactoside

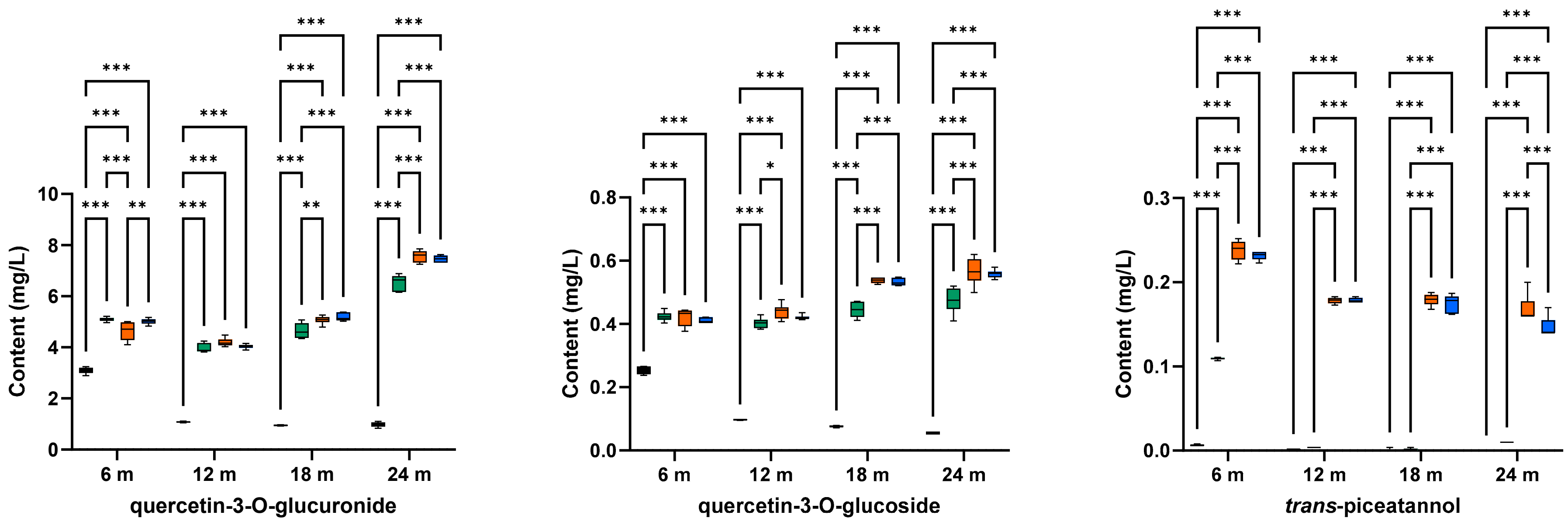
Content (mg/L)
6 m
12 m
18 m
24 m
quercetin-3-O-glucuronide
quercetin-3-O-glucoside
trans-piceatannol

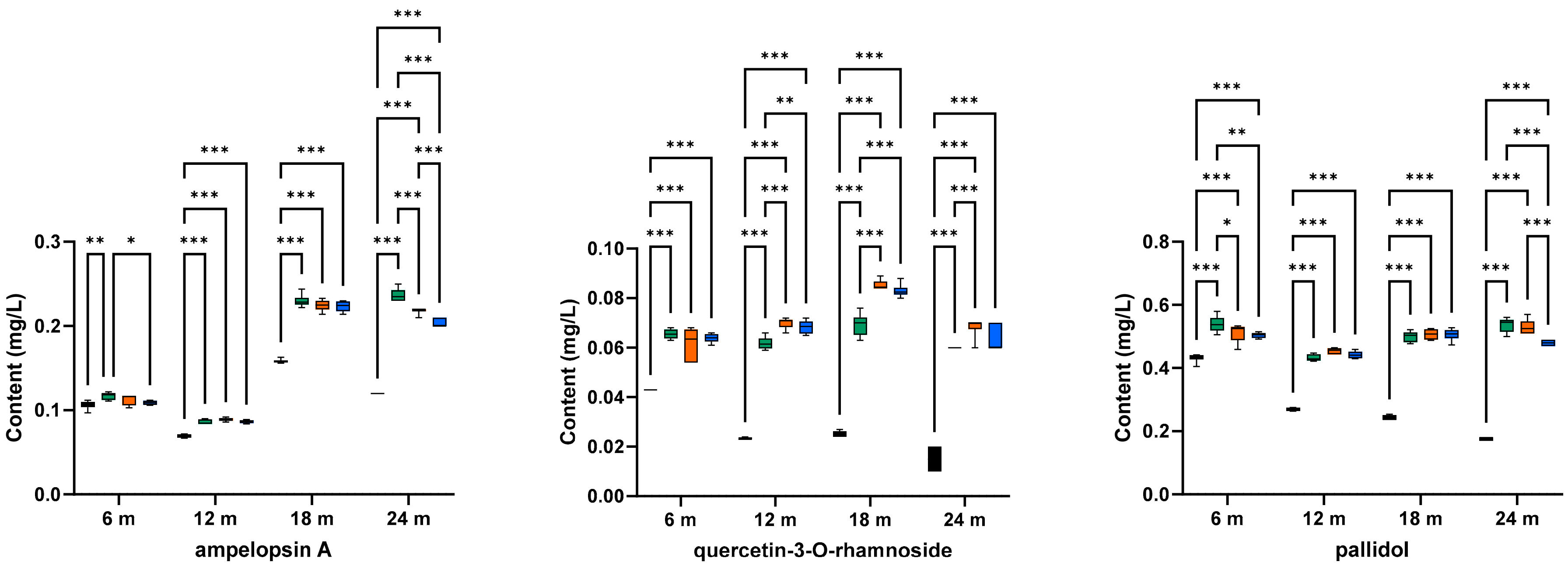
Content (mg/L)
0.0
0.1
0.2
0.3
6 m
12 m
18 m
24 m
ampelopsin A
0.00
0.02
0.04
0.06
0.08
0.10
quercetin-3-O-rhamnoside
0.0
0.2
0.4
0.6
0.8
pallidol

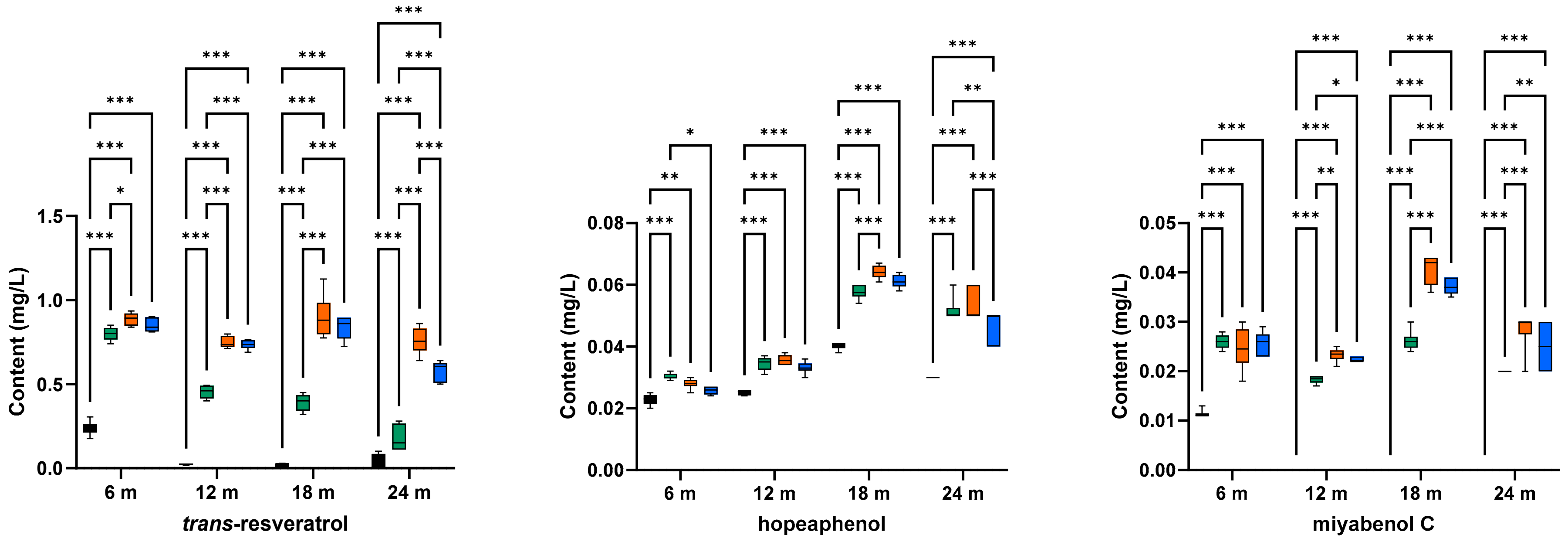
Content (mg/L)
6 m
12 m
18 m
24 m
trans-resveratrol
hopeaphenol
miyabenol C

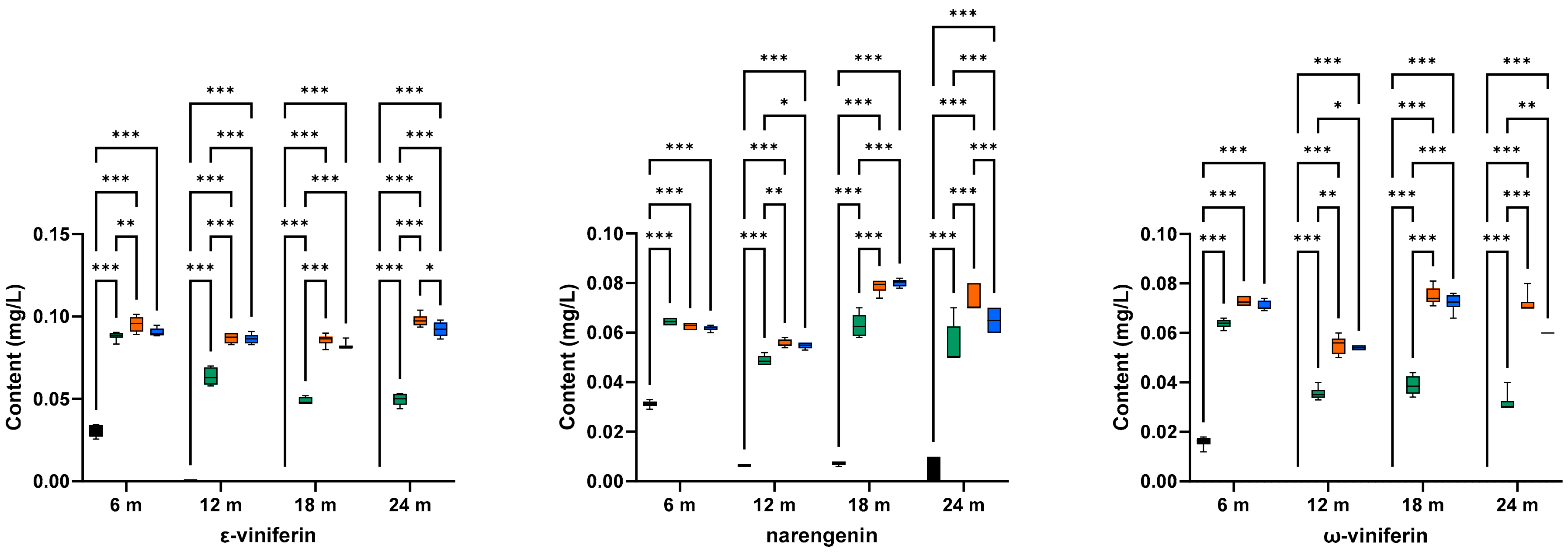
Content (mg/L)
0.15
0.10
0.05
0.00
6 m
12 m
18 m
24 m
ε-viniferin
Content (mg/L)
0.10
0.08
0.06
0.04
0.02
0.00
6 m
12 m
18 m
24 m
narengenin
Content (mg/L)
0.10
0.08
0.06
0.04
0.02
0.00
6 m
12 m
18 m
24 m
ω-viniferin
